# Size-Based Sorting and In Situ Clonal Expansion of Single Cells Using Microfluidics

**DOI:** 10.3390/bios12121100

**Published:** 2022-11-30

**Authors:** Huaying Chen, Haixu Meng, Zhenlin Chen, Tong Wang, Chuanpin Chen, Yonggang Zhu, Jing Jin

**Affiliations:** 1 School of Mechanical Engineering and Automation, Harbin Institute of Technology, Shenzen, Shenzhen 518055, China; 2 Department of Biomedical Engineering, College of Engineering, Kowloon, City University of Hong Kong, Hong Kong, China; 3 Xiangya School of Pharmaceutical Sciences, Central South University, 172 Tongzipo Road, Changsha 410013, China

**Keywords:** size, sorting, separation, clonal expansion, microfluidics, single cells

## Abstract

Separation and clonal culture and growth kinetics analysis of target cells in a mixed population is critical for pathological research, disease diagnosis, and cell therapy. However, long-term culture with time-lapse imaging of the isolated cells for clonal analysis is still challenging. This paper reports a microfluidic device with four-level filtration channels and a pneumatic microvalve for size sorting and in situ clonal culture of single cells. The valve was on top of the filtration channels and used to direct fluid flow by membrane deformation during separation and long-term culture to avoid shear-induced cell deformation. Numerical simulations were performed to evaluate the influence of device parameters affecting the pressure drop across the filtration channels. Then, a droplet model was employed to evaluate the impact of cell viscosity, cell size, and channel width on the pressure drop inducing cell deformation. Experiments showed that filtration channels with a width of 7, 10, 13, or 17 μm successfully sorted K562 cells into four different size ranges at low driving pressure. The maximum efficiency of separating K562 cells from media and whole blood was 98.6% and 89.7%, respectively. Finally, the trapped single cells were cultured in situ for 4–7 days with time-lapse imaging to obtain the lineage trees and growth curves. Then, the time to the first division, variation of cell size before and after division, and cell fusion were investigated. This proved that cells at the G1 and G2 phases were of significantly distinct sizes. The microfluidic device for size sorting and clonal expansion will be of tremendous application potential in single-cell studies.

## 1. Introduction

The heterogeneity of individual cells is widely accepted as an important issue in cell biology and is usually obscured by averaged measurement of large cell populations [1]. Analysis of single-cell growth is critical to understanding the highly dynamic mechanisms that govern cellular plasticity and lineage determination [2,3,4]. However, the traditional method of acquiring single cells in a well plate using limiting dilution has the limitation of low efficiency in both single-cell loading and time-lapse imaging, since only approximately 30% of wells are seeded with one cell per well and the time-lapsed imaging of a 96-well plate in a given time interval has very low throughput in the total number of cells for lineage tracing [5,6]. Benefiting from the size of channels and structures at the microscopic level, microfluidics combined with time-lapse imaging have become powerful tools for long-term clonal analysis of single cells, owing to their advantages in capturing single cells with high throughput and generating a favorable microenvironment.

The study of a small number of cells in a mixed population (e.g., stem cells in umbilical cord blood, tumor cells and megakaryocytes in the circulatory system) plays an important role in pathological research, disease diagnosis, and cell therapy [7,8,9]. In situ culture and time-lapse imaging of target cells isolated from the cell mixture not only save manipulation efforts but also allow detailed investigation of growth dynamics [6,10]. Additionally, in situ culture can avoid the risk of cell loss during recovery and redeposition [11]. For example, a microfluidic device with micropillar arrays was employed to capture CTCs from early-stage lung cancer patients [12]. The captured cells were cocultured with fibroblasts to successfully expand for next-generation sequencing [12]. Moreover, the single cancer cells were trapped in microdroplets and cultured in stem cell media without serum to identify cancer stem cells surviving and forming tumorspheres in the droplets [13].

Label-free capture of single cells is a tremendous advantage of microfluidics for cell analysis [14]. Capture methods such as microwells, droplets, and traps have been developed. These label-free separation methods reduce the preparation time, required reagents, and cost in comparison to traditional methods based on magnetic or immunoaffinity labels [15,16,17,18]. The microwell method typically utilizes cell-sized wells or chambers arranged on the floor of a microfluidic channel to capture target cells by gravity-induced sedimentation [19,20]. However, the low capture efficiency (usually less than 39%) is the major limitation of this technology [21]. Similar to the isolation of individual cells in microwells, another important method for single-cell capture is encapsulating cells in droplets [22,23]. Even though microfluidics can already generate droplets at rates up to 10 kHz, droplets containing single cells follow a Poisson distribution, resulting in lower single-cell encapsulation efficiency and a large number of empty droplets [5,24]. 

Furthermore, hydrodynamic traps generating defined fluid forces have been used to capture single cells in microfluidics [1,22]. For instance, the one-bead-one-compound (OBOC) device [25,26], serpentine-like channels [27,28], and traps with various geometry including cup shape [29,30], U shape [6,31,32], and hook shape [33] were exploited for single-cell capture. However, most of these methods can only capture single cells indiscriminately, while a few of them, such as the u-shaped trap [6], have the potential for the size sorting of cells in mixed populations. Additionally, the small space and large shear stress in these traps are still challenges for in situ clonal culture and time-lapse imaging.

Numerous non-labeling techniques have been exploited to isolate target cells relying on single or multiple physical properties, such as size [34], density [35], electric charges [36], and stiffness [37,38]. Owing to the size overlap between cell types in whole blood, the efficiency and purity of separation based solely on size are dramatically affected. To this end, the stiffness is used as a potential parameter for further sorting of cells [37,38]. For example, pinch flow fractionation (PFF) was used to separate CTCs from white blood cells based on both size and deformability, achieving higher efficiencies for cell size distribution than expected [37]. Although these studies provide substantial insights into efficient cell isolation, most devices are not suitable for in situ long-term culturing and lineage tracing of single cells.

This article describes a microfluidic device for size-based sorting and in situ clonal culture of single cells. Multi-level U-shaped traps not only enabled label-free separation of target cells based on size, but also facilitated long-term clonal culture of captured cells. It has great application potential due to its capability of in situ expansion of cells isolated based on size. The height variability of the culture channel was achieved through membrane deformation, which was beneficial for reducing shear stress on the cells, thereby facilitating cell tiling and lineage tracing. The flow field, pressure drop across the filtration channel, and cell deformation in the device were simulated to evaluate the critical hydrodynamic parameters during both isolation and culture of single cells. The separation efficiency of K562 cells, in both media and whole mouse blood, was characterized. The size sorting capability at various inlet conditions was evaluated. Finally, the captured single cells were cultured for up to seven days with time-lapse imaging to analyze the lineage relations and growth dynamics. This study has significant application potential in the clonal expansion and analysis of rare target cells.

## 2. Materials and Methods

### 2.1. Device Design and Fabrication

**Figure 1:**
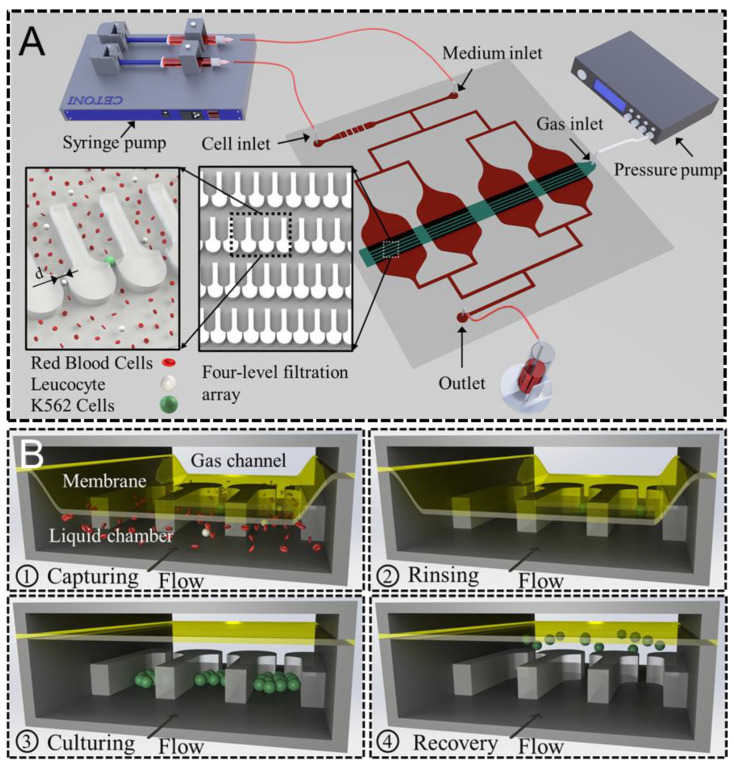
Schematic drawing of the microfluidic system for screening and clonal expansion of circulating tumor cells. (**A**) The whole system including a microfluidic chip containing four-level filtration arrays (inset) with sizes of 17 μm, 13 μm, 10 μm, and 7 μm. (**B**) The membrane of the pneumatic valve was employed to either increase or decrease the fluid shear stress through the filtration channels during capturing/rinsing and culturing/recovery, respectively.

A microfluidic chip (Figure 1A) for sized-based screening and in situ clonal culture of single cells is fabricated by photolithography and PDMS molding as described elsewhere [39]. The chip consists of three layers: a pneumatic layer with a gas channel measuring 1.72 mm × 30 μm (width × height), a 30 μm thick membrane, and a fluid layer. The fluid layer contains four identical chambers (30–100 μm deep) with four levels of filtration microchannels (30–100 μm high) on the floor of each chamber. The widths of the filtration microchannels are 17 μm, 13 μm, 10 μm, and 7 μm from upstream to downstream, respectively. In total, 864 traps are set in the device to capture cells. The varied channel size realizes the capture of cells with different sizes at distinct levels. Moreover, a U-shaped trap (130 × 66 μm) in front of each microchannel is designed to enclose the captured single cells and all daughter cells during long-term culture. 

The pneumatic valve placed on top of all filtration arrays in each chamber is designed to guide the flow by deforming the membrane to the top surface of the filtration arrays (Figure 1B). During capturing and rinsing, all liquid flows through the microchannels to guarantee that each cell of interest is retained by the filtration channel. However, when the membrane is undeflected, most liquid is shunted over the top of the microchannels to reduce the fluid shear stress on the trapped cells and prevent either deformation or loss of cells during the whole culture period. After clonal culture, the undeformed membrane allows all cells to flow over the filtration region to the outlet at higher flow rates and hence realizes the easy recovery of cultured cells.

### 2.2. Numerical Studies of the Flow Field

Both a 2D model of the whole chip and 3D models of one filtration channel with the U-shaped traps were created in COMSOL Multiphysics^®^ (Stockholm, Sweden) to study the pressure drop across the filtration channel (Figure S1 in Supplementary Materials). Models containing only the filtration channel (30–100 μm in height) were developed to evaluate the influence of channel height on the pressure drop. Besides, models consisting of a filtration channel (30 μm high) and a top channel (30–100 μm high) were employed to investigate the impact of the valve membrane on the flow. The inlet ports were supplied with the flow velocities as shown in Table S1 to ensure the equivalent flow rates (at the inlets of the chip) of 1.2–10 μL/min and 2.6 μL/min for the 2D and 3D models, respectively. The outlet pressure was set to zero. All walls were defined as non-slip boundaries.

### 2.3. Numerical Studies of the Cell Deformation

Cells are elastic and may flow through the microchannels during separation owing to the significant deformation induced by the high shear stress. Consequently, 2D models (see Figure S2) combining both the level set and two-phase flow methods were established to study the traversing of single cells with various sizes and viscosities through the filtration channel [6,40]. Liquid droplets with a diameter ranging from 15 μm to 30 μm were employed to model the cells. The viscosity (μ_c_) of the droplet varied from 5 to 40 mPa∙s as the viscosity of cells (leukocytes and cancer cells) is usually considered to be in the range of 1 to 100 mPa∙s [40,41,42]. The interfacial tension coefficient between the droplet and the cell culture media was 50 mN/m [43]. Additionally, blood plasma and cell culture media were set as a Newtonian fluid with a viscosity of 1 mPa∙s [40,41]. The density of cells and plasma/culture media were set to 1025 kg/m^3^ [44,45] and 1000 kg/m^3^ [40,46], respectively. All channel walls were assumed to be non-wetting, with a contact angle of 180°. The inlet velocity in the model was set to 0.04 m/s, which was equivalent to the flow rate of 21.8 μL/min at the inlet port of the chip. The maximum pressure when the cell squeezed through the microchannel was extracted after simulation.

### 2.4. Isolation of K562 Cells in Culture Media

The influence of both the flow rate and the filtration channel depth on the separation efficiency was studied using single-layer devices (without pneumatic valves) containing only the filtration channel with a depth of either 30, 50, or 100 μm. After the device was mounted on a motorized microscope (Olympus IX83, Tokyo, Japan) equipped with a SCMOS camera (Prime 95B, GA, USA), the flow channel system was sterilized for 20 min using 75% (*v/v*) ethanol injected at 30 μL/min. Subsequently, 1% Pluronic F127 solution (Energy Chemical, China) was injected at the same flow rate for 15 min to prime the channels and prevent non-specific cell adhesion. Finally, K562 cell suspension (1 × 10^5^ cells/mL) was loaded from the cell inlet using either a syringe pump (CETONI neMESYS 290N, Korbußen, Germany) at a flow rate of 1.2–10 μL/min or a pressure pump (Elveflow OB1, Paris, France) at 12–20 mbar. After injection for 3 min, the channels were rinsed using cell culture media (loaded from the media inlet) to remove all uncaptured cells throughout the chip. The outlet channel was video recorded during screening and rinsing to record the number (*N_e_*) of escaped cells. Afterward, all U-shaped traps were imaged to obtain the number (*N_c_*) of captured cells. The separation efficiency was calculated by *N_c_/(N_c_ + N_e_)*  × 100%. All experiments in this paper were repeated at least three times unless stated otherwise.

### 2.5. Isolation of k562 Cells in Whole Blood

The ability of the device to separate potential target cells from whole blood was validated using mouse whole blood spiked with K562 cells. Firstly, Hoechst 33342 (Beyotime Biotechnology, Nantong, China) solution was mixed with concentrated K562 cell suspension with a volume ratio of 3:1 and incubated at 37 °C for 30 min. Afterward, the cells were centrifuged at 1500 rpm for 3 min and washed 3 times using PBS before being resuspended to media to a concentration of 1 × 10^6^ cells/mL. Finally, K562 cells were mixed with mouse whole blood (Suyan Biotech, Suzhou, China) with a volume ratio of 1:25 to form a concentration of approximately 40 cells/μL. 

After sterilization of the microfluidic chip, the blood with K562 cells was loaded from the cell inlet at 6 μL/min for a few minutes. Afterward, PBS buffer was injected at the same flow rate from the media inlet for 10 min to flush all blood cells while retaining the K562 cells in the U-shaped traps. Meanwhile, the outlet port was monitored using a fluorescent microscope with a 10× objective to record the escaped K562 cells during separation and rinsing. After rinsing, all traps were imaged using both the phase contrast and the fluorescent modes to acquire the number of captured K562 cells and calculate the separation efficiency. 

### 2.6. In Situ Clonal Expansion of Trapped Single Cells

The trapped single cells were cultured in situ for multiple generations with time-lapse imaging to analyze the growth dynamics. The microfluidic chip with 30 μm deep filtration channels, either with or without a pneumatic valve, was fixed in a stage-top incubator (Tokai Hit STX, Fujinomiya, Japan) on the motorized microscope. The incubator was used to precisely regulate the temperature (37 ± 0.1 °C), concentration of CO_2_ (5%), and moisture (95%). 

After a positive pressure of 2 bar was applied to the gas channel to deform the PDMS membrane, K562 cells were injected and captured by the filtration array using a similar protocol to that mentioned above (Figure 1B Capturing). After the complete rinsing of the chip using media, the membrane was deactivated to the normal state (B rinsing). Then, high-glucose Dulbecco’s Modified Eagle Medium (DMEM) (Sigma-Aldrich, Missouri, USA) with 10% fetal bovine serum (FBS) (JRH Biosciences, Kansas, USA) and 1% penicillin-streptomycin (P-S) (Sigma-Aldrich, Missouri, USA) stored in a 5 mL syringe was continuously injected into the chip at 0.3 μL/min through the media inlet (B culturing) for up to 7 days. Meanwhile, approximately 30 positions with distinct trapped single cells were imaged every 3 min using phase-contrast mode with a 10X objective. The media in the syringe was protected from light at room temperature during the whole culture period.

### 2.7. Cell Lineage Tracking and Data Analysis

The time-lapse image stacks for the clonal expansion of single cells were processed using TrackPad to analyze the cell growth dynamics and obtain lineage trees [47]. TrackPad is open-source, semi-manual software with the ability to track the movement and division of individual cells and create lineage trees of clonal cultured cells [47]. The images for cell separation were processed either using ImageJ [48] or manually to acquire the number of trapped and escaped cells. All error bars in this study represent the standard deviation of at least three replicates. One-way ANOVA and Tukey’s range tests with a significance level of 0.05 were employed to study the differences in the size of cells trapped in different levels.

## 3. Results

### 3.1. Numerical Studies of the Flow Field

**Figure 2:**
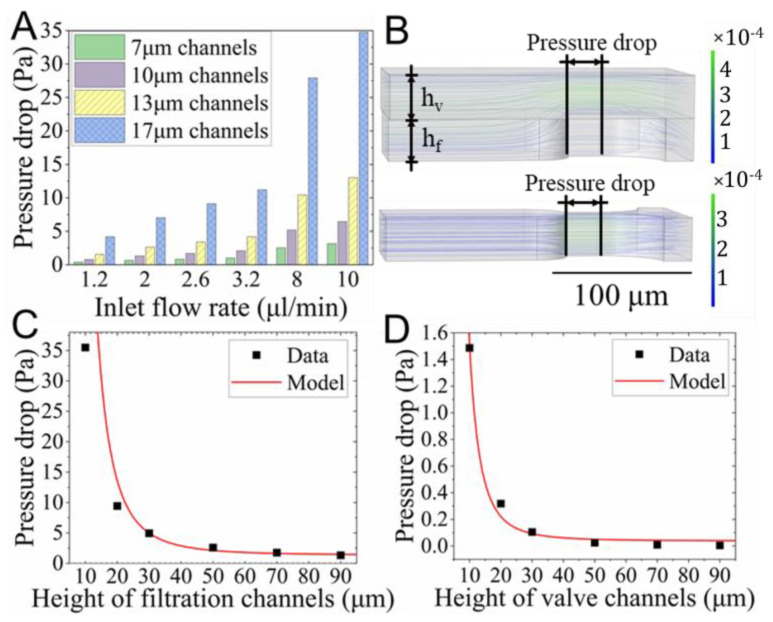
Numerical studies of the flow field. (**A**) The pressure drop across the four-level filtration channels with widths of 7−17 μm at various flow rates. (**B**) The streamlines in models containing a 13 μm wide filtration channel with (**top**) or without (**bottom**) the valve channel at the flow rate of 2.6 μL/min. The *h_v_* and *h_f_* refer to the height of the valve channels and the filtration channels, respectively. The pressure drop at the flow rate of 2.6 μL/min when the height of (**C**) the filtration and (**D**) the valve channels varied.

The flow field in the microdevice without the valve was simulated using 2D models (Figure S1) to analyze the pressure drop across the filtration channels when no cells were captured. As depicted in Figure 2A, for a given channel width, the pressure drop (∆P) was linearly proportional to the inlet flow rate (*Q*). This relationship was described by $∆P\;=\;k_{1}Q\;+\;C_{1}$ (*R*^2^ ≈ 1). Similarly, at the given inlet flow rate, the pressure drop was inversely proportional to the channel width (*w*) and given by $∆P\;=\;k_{2}w\;+\;c_{2}$ (*R*^2^ was between 0.896 and 0.897). The values of $k_{1}$, $k_{2}$, $c_{1}$, and $c_{2}$ at various conditions were listed in Tables S2 and S3. The result illustrates that cells pass through the filtration channels easily due to the higher pressure drop as the flow rate increases.

The impact of the filtration and valve channel was studied using the models indicated in Figure 2B. The top model consisted of a 30 μm deep filtration channel and a valve channel with a height varying from 10 to 90 μm. It allowed the simulation of different deflection stages of the valve membrane, which defined the clearance of the valve channel, while the bottom model represented the case of a fully closed valve. In other words, the fluid flowed only in the filtration channel. The density of the streamlines in the top model demonstrated that most of the fluid was directed to the valve channel. The velocity in the filtration channel was only 0.144 mm/s, which was 3.4 times smaller than that in the valve channel (0.489 mm/s). However, when the valve was completely closed (bottom model), all fluid traveled through the filtration channel, and therefore the velocity (3.825 m/s) was 25.6 times larger than that in the former case. 

As depicted in Figure 2C,D, the pressure drop (∆P) was inversely related to the height of both the filtration ($h_{f}$) and valve ($h_{v}$) channels. The relationship was given by $∆P\;=\;97860/h_{f}^{3}\;+\;1.35\;$($R^{2}\;=\;0.987$) and $∆P\;=\;1460/h_{v}^{3}\;+\;0.03875\;$($R^{2}\;=\;0.992$). For example, when the height of the filtration channel increased from 10 μm to 30 μm, the pressure drop dramatically decreased from 35.5 to 4.94 Pa. Similarly, for the 30 μm deep filtration channel, the pressure drop was 4.94, 0.105, and 0.00422 Pa for the valve channels with heights of 0, 30, and 90 μm, respectively. This suggested that the valve channel significantly reduced the pressure drop due to its flow-shunting effect. 

The pressure drop across the filtration channel is the most critical parameter affecting the separation efficiency and in situ retention of cells during clonal culture. Detailed study may help one to optimize the geometry and control parameters of the microfluidic chip. To increase the throughput of cell separation, a higher inlet flow rate is preferable, whereas pressure drop at a higher flow rate may induce undesirable cell loss due to deformation, since the elasticity of cells is only dozens to thousands of Pascal [47]. Additionally, during clonal culture, the flow of perfused media may induce cell deformation and even loss when the pressure drop is big enough. Thus, the aforementioned findings are essential to optimize the inlet flow rate, channel height, and valve state to maintain the pressure drop within a reasonable range. Moreover, the pneumatic valve in this study may dynamically adjust the pressure drop to not only increase the separation throughput but also minimize the cell loss during clonal culture.

### 3.2. Numerical Studies of the Cell Deformation

**Figure 3:**
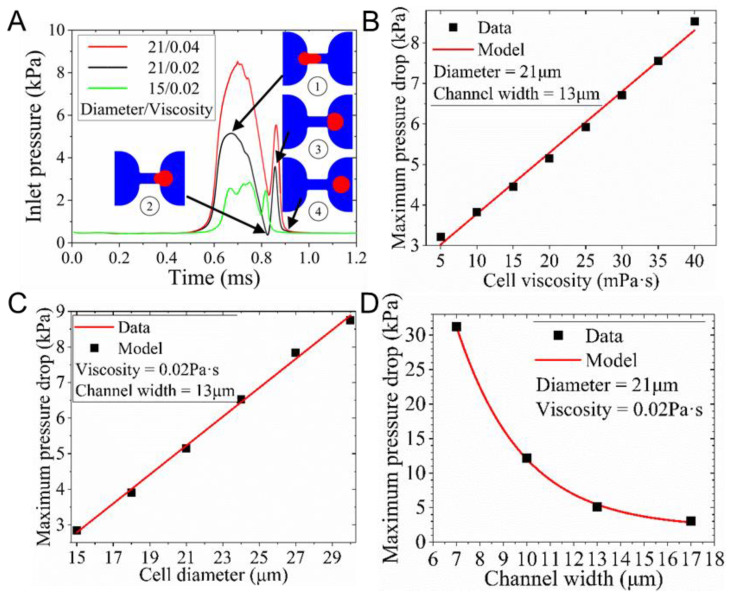
Studies of a cell passing a filtration channel modeled using a droplet. (**A**) The variation of the pressure drop inducing the deformation of cells with distinct sizes and viscosities at the inlet velocity of 0.04 m/s. The relationship between the maximal pressure drop and (**B**) the viscosity, (**C**) cell diameters, or (**D**) channel width.

Figure 3A demonstrates the variation of pressure drop (between the inlet and outlet of the model) causing the deformation and traversing of a cell through a 13 μm channel. When the cell deformed and protruded into the microchannel for some distance (Figure 3A①), the pressure drop reached the first peak (maximal value). When it left the channel outlet (Figure 3A②), the pressure drop returned to its trough. The third peak suggested the additional force deforming the droplet further when it flowed away from the channel outlet (Figure 3A③). These findings were consistent with previous studies using a droplet to model a cell [41]. 

The 15–30 μm diameter droplets used in numerical studies were based on the sample size in the experiments, as the smallest K562 cell captured using this device was 13.7 μm in diameter. The size of individual cells may increase to 25.1 μm (the cell size before division) during cell culture. Figure 3B–D suggests that the maximum pressure drop ($∆P_{max}$) was linearly proportional to both the viscosity ($\mu_{c}$) and the size ($R^{2}\;=\;0.994$), $∆P_{max}\;=\;0.407d\;-\;3.311\;$($R^{2}\;=\;0.997$), and $∆P_{max}\;=\;356.7\;\times \;\exp\left(-\frac{w}{2.797}\right)\;+\;2.044\;$($R^{2}\approx 1$). The intercepts in the equations indicated the pressure drop across the channel without cells. For a 21 μm cell, when the viscosity rose from 5 to 40 mPa∙s, the maximum pressure drop increased merely 2.6 times. However, it rose 3.1 times when the cell diameter doubled from 15 to 30 μm. While the width of the microchannel decreased from 17 to 7 μm, the maximum pressure for a 21 μm cell to pass through the channel rose dramatically to 10.3 times. This reveals that the influence of both the channel width and the cell size on the pressure drop is much more significant than that of the cell viscosity.

The viscosity of cells varies widely because the viscosity of the internal fluid is an apparent viscosity affected by the cell structure and the substance in the cell. The viscosity of cells (leukocytes and cancer cells) in previous numerical simulations is considered to be in the range of 1 to 100 mPa∙s [40,41,42]. Therefore, it is reasonable to employ a viscosity ranging from 5 to 40 mPa∙s in the numerical studies. The maximum pressure corresponding to the flow extrusion through the 13 μm wide channel is between 2.85 kPa and 8.75 kPa, according to the aforementioned models. However, the real pressure drop to drive K562 cells and endothelial cells through a 6 μm (width) × 16 μm (height) channel was in the range of 175–925 Pa and 550–1750 Pa, respectively [49]. This suggested that the pressure-inducing cell traversing through a microchannel was much smaller than predicted in this study. 

This drastic difference may be related to the droplet model and its boundary conditions. Firstly, the 2D model cannot investigate cell deformation in the third direction. Moreover, the model assumes a cell to be a perfect droplet with uniform viscosity. However, the cell is a viscoelastic sphere with a cell membrane and nucleus, which were ignored in the droplet model. However, the simulation can still allow one to quantitatively evaluate the influence of device geometry on the pressure drop so that the device can be optimized to avoid cell deformation or to pass through the channel during separation.

### 3.3. Isolation of K562 Cells from Culture Media

**Figure 4:**
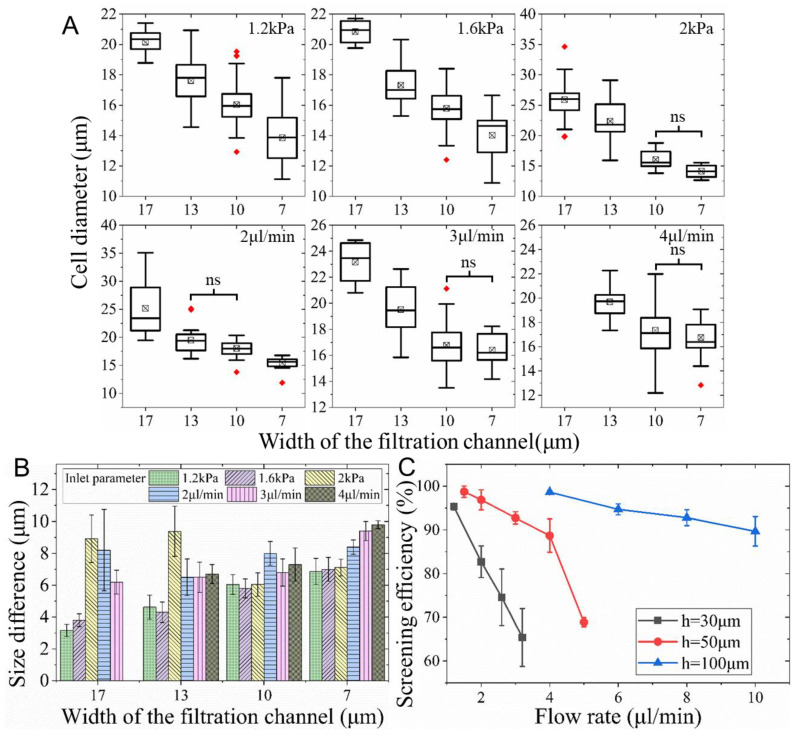
Size-based screening of K562 cells. (**A**) The size of cells (4 < N < 93) trapped by each filtration level and (**B**) the difference of cell size and filtration channel width when cell suspension was injected at various pressure or flow rate. (**C**) The separation efficiency of filtration channels with various heights (h) at distinct flow rates.

The sizes of cells isolated by four filtration levels were characterized to demonstrate the ability of the device to screen cell size. As depicted in Figure S3, the diameter of K562 cells in normal culture was normally distributed, with a mean (±standard deviation) of 17.8 (±2.8) μm. The boxes in Figure 4A show the size of cells trapped by each level when a cell suspension was injected at either a given pressure or a specific flow rate. The central lines, bottom/top edges, and the whiskers represent the median, 25%/75% percentiles, and most extreme data not considered as outliers, respectively. The cross boxes and red diamonds indicate the mean and outliers, respectively.

Figure 4A suggested that the four-level filtration arrays were capable of capturing cells with different size ranges. According to Tukey’s tests, cell sizes in the four filtration levels were significantly different from each other at inlet pressures of 1.2 and 1.6 kPa, whereas cell sizes between the 10 μm and 7 μm levels had no significant difference at an inlet pressure of 2.0 kPa. This demonstrates that pressure lower than 1.6 kPa is favorable to guarantee the ability of the device to screen cell sizes with significant differences. When the inlet pressure was 1.2 kPa, the mean diameters of cells trapped by 17 μm, 13 μm, 10 μm, and 7 μm microchannels were 20.2 μm, 17.6 μm, 16.0 μm, and 13.7 μm, respectively. As the inlet pressure increased from 1.2 to 2 kPa, the size of the cells trapped by 17 μm and 13 μm microchannels was augmented by 28.2% and 27.3%, respectively. This suggests that cells that can be trapped by wider channels at lower pressure may be deformed considerably and escape downstream at a higher pressure. Thus, the same wider channels can only capture larger cells at higher flow rates. However, the size of cells captured by the 10 μm and 7 μm microchannels did not change significantly (*p* = 0.46 and 0.93). This is because the pressure augmentation fails to induce cell loss due to deformation since, as discussed in the simulation section, much higher pressure is required to deform cells through narrower channels. The smallest average size of K562 cells successfully captured using this device was 13.7 μm, correlating with cell diameter and elasticity. Consequently, target cells with different sizes and stiffness can be captured appropriately with specific filtration channel size, inlet pressure, and flow rate.

When cells were injected at the given flow rate, the device was also capable of screening cell size, whereas the sizes in-between the 10 μm and 13 μm (between 10 μm and 7 μm) filtration levels at 2 and 3 μL/min (at 4 μL/min) had no significant difference. Therefore, it can only screen cell size without notable overlap between every other filtration level. This screening capability was worse than the case with a given inlet pressure since the pressure drop across filtration channels was fixed and accurately regulated. Nonetheless, for a given inlet flow rate, when some microchannels are blocked by cells, the pressure drop may vary significantly, causing considerable cell deformation/loss and hence affecting the screening accuracy.

Additionally, Figure 4B depicts the difference between the mean cell size and the relevant filtration channel width. Size difference rose as the filtration channel became narrower. For instance, it was 3.15, 4.62, 6.04, and 6.87 μm for the 17, 13, 10, and 7 μm wide channels, respectively, at the inlet pressure of 1.2 kPa. One-way ANOVA revealed that the size difference for the same filtration channel had no significant difference when the inlet pressure was 1.2 and 1.6 kPa (*p* = 0.15–0.77). Moreover, experiments with given flow rates were of a bigger size difference than those with given inlet pressure. These findings further proved that separation driven with a pressure that was not larger than 1.6 kPa was optimal for size-based screening of cells using this device. 

The size difference was raised approximately from 3.15 to 9.8 μm as the channel width and inlet condition varied. The size difference is mostly related to cell elasticity, channel width, and the pressure drop across the filtration channel. Since the elasticity of K562 cells was normally in the range of dozens to hundreds of Pascals [49], the cells can easily deform and squeeze through the filtration channels. Thus, the trapped cells are usually a few microns bigger than the channel width. 

Further experiments proved that employing higher filtration channels can not only improve the separation efficiency, but also enhance the separation throughput by using a higher flow rate. Figure 4C revealed that the separation efficiency drastically decreased with the rise of flow rates regardless of the channel height. For example, it dropped from 98.7% to 68.9% when the flow rate increased from 1.5 μL/min to 5 μL/min in devices with 50 μm deep microchannels. This is consistent with a previous study using microstructures and is a result of cell loss due to large shear stress [50]. However, at the given flow rate, the separation efficiency dramatically increased with the rise of the channel height. For instance, at the flow rate of 4 μL/min, it was smaller than 50%, 88.7%, and 98.6% for the channels with heights of 30 μm, 50 μm, and 100 μm, respectively. The prominent improvement in separation efficiency was a result of the pressure decrease as the channel height increased.

### 3.4. Screening of K562 Cells from Whole Blood

**Figure 5:**
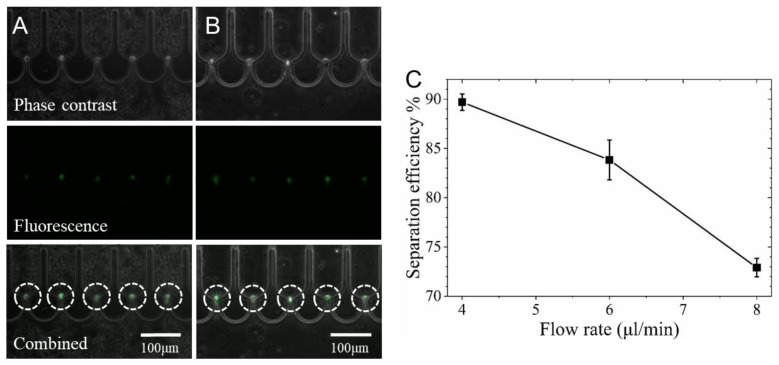
The separation of K562 cells in mouse whole blood using a microdevice with 100 μm deep filtration channels. The phase contrast, fluorescent, and combined images of isolated K562 cells (highlighted in dotted circles) (**A**) in whole blood and (**B**) in PBS after rinsing of blood cells at 4 μL/min. (**C**) The separation efficiency at various inlet flow rates.

The ability to screen rare target cells from whole blood was verified using a microfluidic chip with 100 μm high microchannels. Mouse blood was employed to model human blood since the cells of both were of very similar sizes (Table S4). As shown in the Supplementary Video S1, after isolation of individual K562 cells in the U-shaped traps (Figure 5A), the blood cells could still pass through the same channel since their sizes were much smaller than that (17.8 ± 2.8 μm) of K562 cells. After rinsing using PBS at 4 μL/min, no obvious loss of K562 cells was observed (Figure 5B). As shown in Figure 5C, the separation efficiency decreased from 89.7% to 73% when the flow rate of blood was raised from 4 to 8 μL/min. Therefore, the device with 864 traps can theoretically process 24.1 μL of mixed blood (40 K562 cells/μL) at a flow rate of 4 μL/min in 6 min based on the 89.7% capture efficiency. The efficiency was 8.9–19.9 % smaller than that of separating K562 cells in media at the same flow rates (Figure 4C). 

Rare target cells were modeled using K562 cells spiked in mouse whole blood with a concentration of 40 cells/μL. In comparison to red blood cells (10.6 million/μL) and white blood cells (2.6 k/μL), the concentration of K562 cells in whole blood was reasonable for the study of rare cells. Additionally, the isolation from mixed mouse blood containing large amounts of non-target particles, such as red blood cells, can reflect the ability of the device to work in a non-Newtonian fluid (blood).

In the experiment, only a few mouse white blood cells (WBCs) were found among over five hundred captured cells, although the number of WBCs was 65 times that of the K562 cells in the mixed whole blood. This result could be caused by the difference in the size of cells. Since elastic cells may deform and squeeze through the filtration channels, most of the traps with 7–17 μm wide channels captured K562 cells with a minimal size of 16.7 μm at the flow rate of 4 μL/min (Figure 4A). This is over twice the width of the smallest filtration channels. Therefore, WBCs with sizes of approximately 10–15 μm [51] were unlikely to be captured at the flow rate of 6 μL/min, although some of them were larger than the smallest filtration channel (7 μm) since they might easily squeeze through the channels after deformation.

The reduction in separation efficiency is probably related to the liquid property difference between media and whole blood. Blood is a non-Newtonian fluid with a viscosity of 3.5–5.5 mPa·s. As a result, the pressure drop across the filtration channel with blood may be significantly higher than that of media, even at the same inlet flow rate. The cell loss due to deformation in blood flow is much higher and thus dramatically reduces the separation efficiency.

### 3.5. In Situ Clonal Expansion of Trapped Single Cells

Following the isolation from media, K562 single cells were cultured in situ for at least one week in devices either with or without a pneumatic valve to reduce flow shear stress. As shown in Supplementary Video S2, the U-shaped traps provided sufficient space for the captured cells and their progenies, so all cells from the same clone cultured for one week were successfully retained in situ for time-lapse imaging. As depicted in Figure S4A , the cells were mostly stacked together to form multiple layers due to the large shear stress in devices without valves, whereas adherent cells can naturally form a monolayer on the bottom of the chamber [6]. This multilayer is detrimental for lineage tracking since individual cells cannot be recognized from the image. In contrast, the opening microvalve effectively shunted most flow over the top of the filtration channel and thus reduced the shear stress on cells. Therefore, all cells formed a monolayer on the floor of the U-shaped trap (See Figure S4B), which enabled the identification of the division and lineage relations of each cell from the image stacks. 

**Figure 6:**
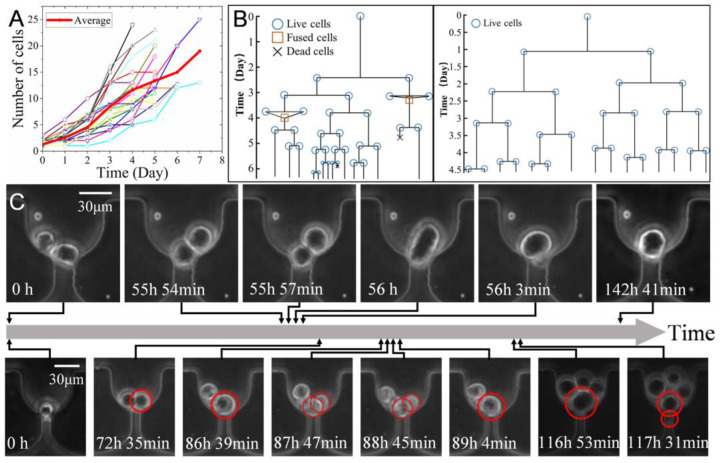
In situ clonal expansion of captured K562 cells. (**A**) The growth curves of 22 individual clones within 7 days. The line with dots shows the average of all clones. (**B**) The lineage trees of two clones. The line length suggests the division time. (**C**) Micro photos showing the one cell divided into two cells, which were fused into one cell dividing into two daughter cells later.

After semi-automatic processing of the image stacks using TrackPad [47], the motion and division of all cells were recorded for the analysis of both growth dynamics and pedigree. In comparison to our previous study showing the isolation and clonal culture of single cells using microwell arrays, the current study mainly focused on the performance in size screening and the cell growth dynamics in detail (e.g., cell fusion, cell size variation, etc.) [6]. Over 4–7 days of in situ culture, 46.2% of the trapped single cells (N = 119 cells) remained alive and successfully divided at least once; of these, 52.7% expanded to clones. Figure 6A illustrates the growth curves of 22 individual clones over 4–7 days. On day 7, the original mother cells expanded to 13–25 progenies in each clone. Thus, the cell doubling times were 32.3 ± 7.5 and 40.8 ± 4.6 h [6] for 4 and 7 days, respectively. This is longer than the doubling time (26 h) of cells cultured as populations in flasks [52] but smaller than that (46 ± 5.3 h) of single K562 cells cultured using a microwell device [6]. This difference in doubling time might be related to the difference in both the passage number and the microenvironment on the chip. Nevertheless, it suggested that this culture system was able to replicate as similar growth conditions as possible in comparison to the normal population-based culture. 

The lineage trees of two clones with 6–7 generations in Figure 6B shows the time of division, fusion, and death of cells. The time to the first division for each trapped single cell (N = 19) ranged from 4 to 39 h (with the mean ± std of 13.46 ± 3.89 h). It was largely related to the cell cycle when they were trapped for culture since the suspended cells barely required time to adhere to the surface and fit the microenvironment as adherent cells do. Additionally, the division times (vertical lines in Figure 6B) of cells even in the same generation were significantly different. This not only suggested heterogeneity at the single-cell level but also highlighted the significance of performing statistical analysis.

Automatic fusion of two daughter cells was observed in the experiment (see Figure 6B,C). Two cells can be fully fused in 9–85 min (N = 6). The fused cells in Figure 6B are divided into two, and this rarely occurs in normal culture conditions [53]. It is usually induced by chemicals [54], virus [55], or electrical pulses [56]. Microstructures and flow-induced deformation were employed to increase cell fusion efficiency in microfluidics [53]. Similarly, the frequent occurrence of cell fusion in this experiment is probably due to the physical confinement and flow-induced formation of cells in the U-shaped trap. However, more experiments with statistical significance are necessary to further reveal this phenomenon.

**Figure 7:**
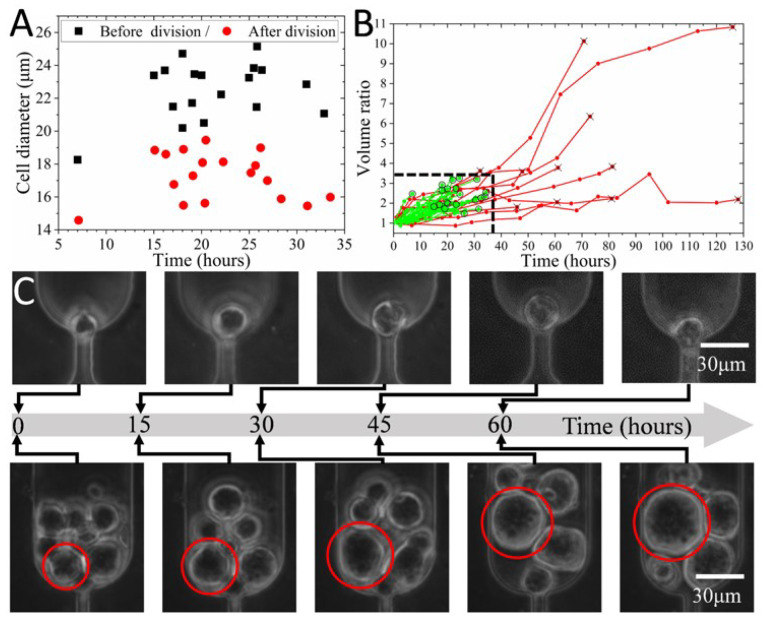
The size variation of different cells during culture. (**A**) The size of cells immediately before and after division (N = 18). (**B**) The variation of cell volume ratio during culture (N = 33). Green lines with circles and red lines with crosses indicate the cells with or without division, respectively. (**C**) The micro photos showing cells without division with either negligible (**top**) or 10.1 times (**bottom**) volume increase in 60 h.

Time-lapse imaging enables detailed study of cell size variation during cell growth. A previous study reported that the size of cells in the same clone decreased by 30% immediately after division [6]. This study further proved that the mean (±std) size of a cell before and after the division was 22.47 (±1.75) μm and 17.2 (±1.47) μm, respectively. Figure 7A indicated that cells were divided into two groups with significantly distinct size ranges. The bigger cells were at the late M phase and about to divide, while the smaller ones represented daughter cells at the beginning of the G1 phase. For the same cell, as growth and DNA replication occurred, its size dramatically increased by 30.5 ± 6.2% (N = 18) at the end of the G2 phase. 

It is reasonable to conclude that the size difference in the cell population reflects not only the heterogeneity but also the cell cycle. Therefore, it is possible to sort the cell cycles based on cell sizes [57] using this device. The aforementioned section has proved the capability of the device to separate cells into four size groups, including 20.2 μm, 17.6 μm, 16.0 μm, and 13.7 μm. This size range covers the mean size of cells before and after division. Consequently, this device has the potential to be used for cell cycle synchronization based on size [49].

In addition to size before and after division, the variation of cell volume with culture time was studied based on the time-lapse image stacks. The cell volume rose during DNA replication and reached its maximum just before division. As depicted in Figure 7B, the mean volume ratio and time for the division for cells (N = 21) with dividing capability were 2.4 (±0.5) and 23.4 (±6.9) hours, respectively. However, some cells might not increase their size significantly but stay undivided for 45–80 h (Figure 7B and C top). Alternatively, some cells continuously grew to a much bigger size (e.g., the volume ratio of 10.1) over more than 70 h (Figure 7B,C bottom). Figure 7B revealed that cells with a volume ratio and division time larger than 3.3 and 34.4 h might eventually lose their division capability.

## 4. Conclusions

This paper reports a microfluidic device for size-based sorting and in situ lineage tracking of rare target cells. Flow simulation suggested that the pressure drop across the filtration channels was inversely related to the height of the filtration and valve channels. The pneumatic valve was able to effectively shunt flow to reduce the pressure drop, which may induce cell deformation. Then, a droplet deformation model demonstrated that the cell viscosity, cell size, and channel width were critical parameters determining the pressure provoking cell deformation and traveling through the filtration channel. 

Afterward, experiments showed the ability of the device to screen cells with four distinct size range at driving pressures of 12 and 16 kPa. The separation efficiency of separating cells was inversely related to both channel height and flow rate. The efficiency separating K562 cells from media and mouse whole blood reached maximums of 98.6% and 89.7% at flow rates of 1.5 and 4 μL /min, respectively. Finally, the trapped single cells were cultured for 4–7 days for lineage tracking, with a division rate of 46% and a successful clone formation rate of 53%. In addition to lineage trees of up to nine generations, the growth dynamics, including first division time (23.4 ± 6.9 h) and doubling time (32.6 ± 6.4 h), were acquired. Cell sizes immediately before and after division were found to be distributed in two significantly different ranges, which highlighted the possibility of using the size screening ability of the device to sort cell cycles. This study not only proves the capabilities of the device in size sorting and lineage tracking of rare target cells but also provides the growth dynamics of K562 cells cultured at a single-cell level.

## Data Availability

Not applicable.

## Supplementary Materials

The following supporting information can be downloaded at: https://www.mdpi.com/article/10.3390/bios12121100/s1, Figure S1: 2D flow simulation models; Figure S2: simulation model of single cell traversing through filtration channel; Figure S3: the diameter distribution of K562 cells used in this study; Figure S4: cell images after 5 days culturing, without or with an opening valve on top of the filtration channel; Table S1: inlet parameters of 2D and 3D flow simulations; Table S2: the parameters of linear models describing the relationship between pressure drop across the filtration channel and the flow rates; Table S3: the parameters of linear models describing the relationship between pressure drop across the filtration channel and the channel width; Table S4: size comparison between human and mouse blood cells.
