# Supplementary material for: Size-Based Sorting and In Situ Clonal Expansion of Single Cells Using Microfluidics
Source: Biosensors (Basel). 2022 Nov 30;12(12):1100. doi: 10.3390/bios12121100 (PMC9775143; doi:10.3390/bios12121100)
Supplement: Supplementary file 1 [file biosensors-12-01100-s001.zip › biosensors-2048129-ESI-supplement for xml.pdf]

Experiment

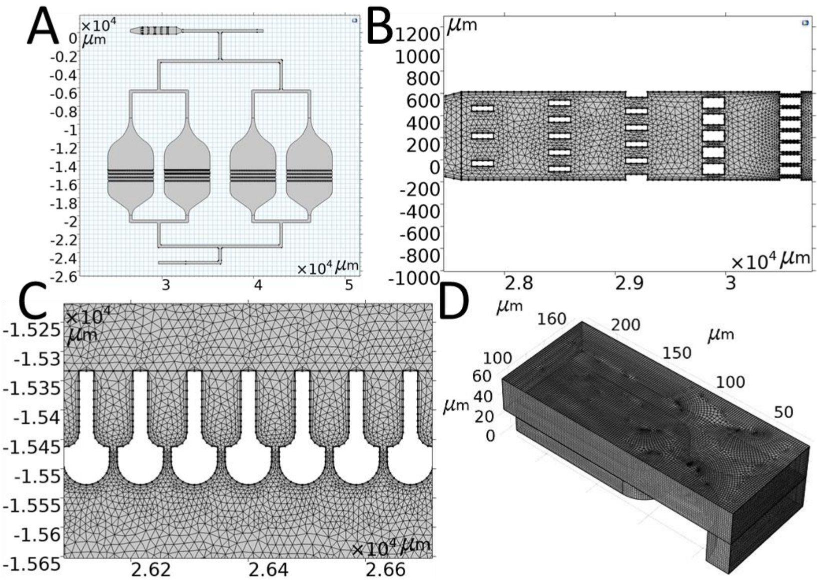

Figure S1. 2D flow simulation models of (A) the whole chip, (B) the inlet area and (C) the filtration channel. (D) 3D flow simulation model of the filtration area.

Table S1. Inlet parameters of 2D and 3D flow simulations.

	Height of filtration channel (μm)	Height of top channel (μm)	Flow rate (μl/min)	Velocity (m/s)
2D models	30	0	1.2	0.0022
			2	0.0037
			2.6	0.0048
			3.2	0.0059
			8	0.0147
			10	0.0183
3D models	30	10	2.6	0.0156
		20		0.0119
		30		0.0096
		50		0.0069
		70		0.0054
		90		0.0044
	10	0		0.0688
	20			0.0344
	30			0.0229
	50			0.0138
	70			0.0098
	90			0.0076

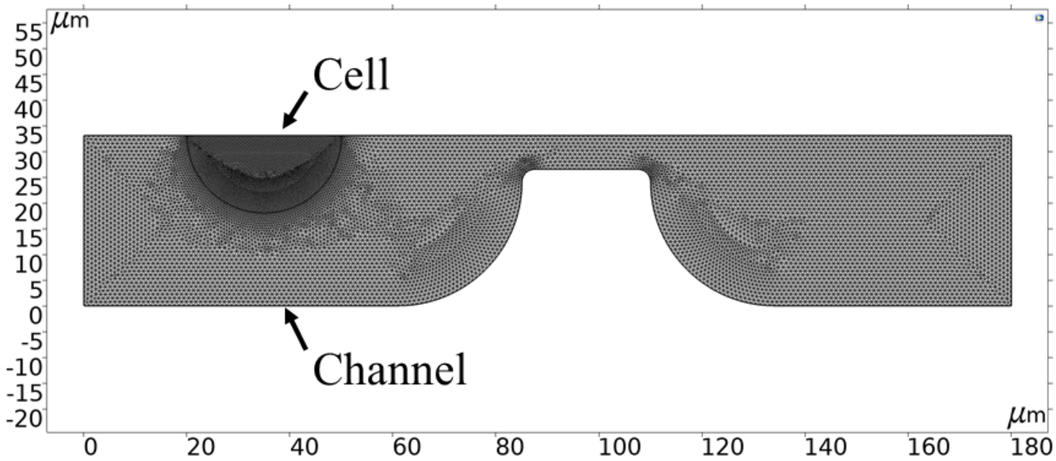

Figure S2. Simulation model of single cell traversing through filtration channel.

Table S2. The parameters of linear models describing the relationship between pressure drop across the filtration channel and the flow rates .

Flow rates (µl/min)	k_1	c_1
1.2	0.378	2.72
2	0.636	4.58
2.6	0.825	5.937
3.2	1.015	7.3
8	2.531	18.22
10	3.152	22.7

Table S3. The parameters of linear models describing the relationship between pressure drop across the filtration channel and the channel width.

Channels width (µm)	k_2	c_2
7	0.314	0.0092
10	0.644	0.0154
13	1.301	0.0163
17	3.476	0.0668

Results and discussion

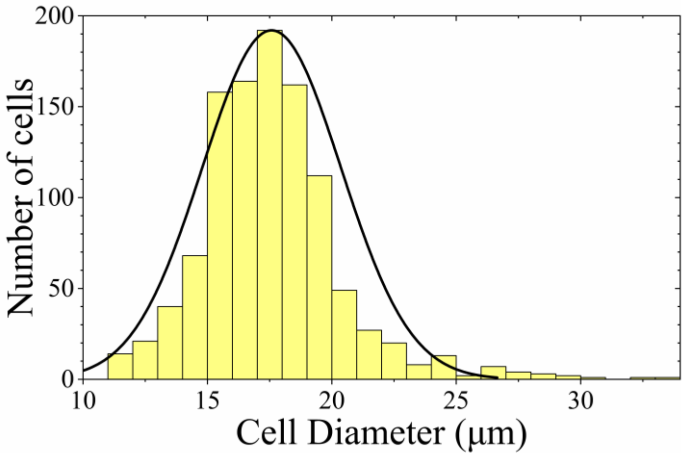

Figure S3. The diameter distribution of K562 cells used in this study

Table S4. Size comparison between human and mouse blood cells[1]

	Diameter of red blood cells (μm)	Diameter of white blood cells (μm)	Diameter of platelets (μm)
Human	5~7	7.3~13.2	1~4
Mouse	4~7	10~15	1~4

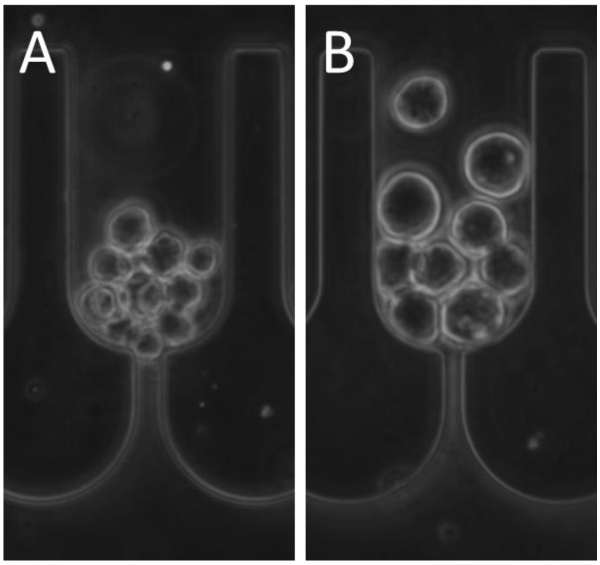

Figure S4. Cell images after 5 days culturing (A) without or (B) with an opening valve on top of the filtration channel.

References

1. *K. E. O'Connell, A. M. Mikkola, A. M. Stepanek, A. Vernet, C. D. Hall, C. C. Sun, E. Yildirim, J. F. Staropoli, J. T. Lee and D. E. Brown, Comp Med, 2015,* ***65****, 96-113.*
